# Active Liver Bleed Caught During FAST Exam from Spontaneous Hemangioma Rupture: A Case Report

**DOI:** 10.5811/cpcem.21315

**Published:** 2025-01-28

**Authors:** Raul Rodriguez, Nicole Aviles

**Affiliations:** HCA Florida Kendall Hospital, Department of Emergency Medicine, Miami, Florida

**Keywords:** case report, FAST, liver, hemangioma, rupture

## Abstract

**Introduction:**

This case highlights the advances that have been made when skilled sonographers using point-of-care ultrasound (POCUS) are able to evaluate for more than free fluid on the focused assessment with sonography in trauma (FAST) exam. Specific solid organ injury including an active liver bleed can also be detected during FAST exam, as seen in this case of a unstable hypotensive patient.

**Case Report:**

A 55-year-old male who had recently been admitted to trauma service due to multiple rib fractures presented back to the emergency department (ED) due to an episode of syncope and was found to have an acute, left segmental pulmonary embolism. The patient was started on anticoagulation, and the following day was found to be hypotensive, encephalopathic, and minimally responsive to verbal stimuli. During the resuscitative efforts, a FAST exam performed by the emergency physician showed grossly positive free fluid in various quadrants and active flow around the liver concerning for active bleeding. Computed tomography subsequently confirmed an active subcapsular bleed of the liver, and patient was taken emergently to surgery for hemostasis from a ruptured liver hemangioma. This was then followed by a right hepatic arterial embolization.

**Conclusion:**

While the FAST exam is well established in the setting of trauma, this case further highlights the use of POCUS in a patient with undifferentiated hypotension and shock. It serves as a reminder of how imperative it is to not anchor on the primary diagnosis and reinforces the importance of ultrasonographic competency in physicians of all specialties and not just those in the realm of emergency medicine and critical care.

## INTRODUCTION

The focused assessment with sonography in trauma (FAST) exam was incorporated into Advanced Trauma Life Support in the 1980s and is still a vital tool in the process of assessing for free fluid specifically in patients who present with traumatic injuries to the region of thorax and abdomen.^1^ Although we are aware of the importance of the FAST exam is critical in traumatic patients presenting with shock and hypotension, it plays a significant role in non-traumatic patients as well.

Medical training in the United States over the last decade has incorporated more point-of-care ultrasound (POCUS) as part of residents’ daily practice. In emergency medicine, critical care, anesthesia, and across most specialties there is a role for POCUS in the right clinical setting for certain patients, and it can lead to a more expedited diagnosis, management, and definitive care.

The utility of the FAST exam has always been an adjunct to quickly and reliably help physicians to assess for hemoperitoneum and hemopericardium.^2^ While evaluation of specific solid organ injury is a known limitation of ultrasound, skilled operators can detect more than just free fluid during the FAST exam. In this unique case our emergency physicians was able to catch, in real time, active color flow noted in the right upper quadrant consistent with what was later confirmed by computed tomography (CT) as an active liver bleed.

## CASE REPORT

Our patient was a 55-year-old male who had been admitted to our trauma service a day prior for pain control and observation after a motor vehicle crash. He had suffered a left seventh and eighth anterior rib fracture and presented on the same day of discharge back to the emergency department (ED) due to an episode of syncope. The patient denied any new symptoms, new trauma, or any changes to his rib pain where the fractures had occurred. Workup in the ED found the patient to have an acute segmental non-occlusive left pulmonary embolism (PE). The patient was subsequently started on anticoagulation with a heparin drip and readmitted to inpatient service for monitoring in the setting of this new acute PE along with recent history of syncope.

The following day a rapid response was called on the patient’s floor by a nurse as the patient was found to be hypotensive, encephalopathic, and minimally responsive to verbal stimuli. The physical exam revealed a tender abdomen and very cool extremities. He was emergently transported for computed tomography (CT) but in the process of transport became increasingly more hypotensive and unstable. At that point the patient was diverted to a resuscitative bay in the ED, and a FAST exam was performed by an emergency physician. The exam was significant for grossly positive free fluid in various quadrants of the abdomen. Furthermore, the emergency physician noted an irregular pattern in the motion of free fluid along with varying echogenicity seen by the liver parenchyma. This promptly the physician to add color doppler to that area of the liver, where an active flow was visualized (as seen in our video--supplemental material), concerning for potential active liver bleed.

The patient was stabilized, given two liters of crystalloids along with blood products, and intubated for airway protection. Radiology and CT of abdomen and pelvis exam confirmed a subscapular hyperdense blush along the right lower hepatic lobe. This was favored to be an active venous subscapular bleed of the liver with new hemoperitoneum from intraparenchymal hematoma given timing of contrast phase (Image). These findings were consistent with what the emergency physician was able to visualize with POCUS in the resuscitation bay.

New laboratory testing on the patient showed a steep drop in hemoglobin from 12.3 grams per deciliter (g/dL) (reference range 13.8–17.2 g/dL) on arrival to 9.0 g/dL along with a new elevated lactic acid level of 7.7 millimoles per liter (mmol/L) (0–2 mmol/L).

**CPC-EM Capsule**

What do we already know about this clinical entity?

*Liver hemangioma rupture and bleeding is more prominent in tumors larger than 4 centimeters and can quickly lead to hemorrhagic shock and death*.

What is the major impact of the image(s)?

*These images provide evidence that specific organ injury and active bleeding could be visualized during a focused assessment with sonography in trauma exam if there is clinical suspicion for it*.

How might this improve emergency medicine practice?

*This case reinforces the usefulness of point-of care-ultrasound in undifferentiated shock and the need to keep a broad differential for all crashing patients*.

The patient was emergently taken to the operative suite where he underwent exploratory laparotomy for evacuation of intrabdominal hematoma, hepatorrhaphy, electrocautery of liver for hemostasis, intrabdominal packing of liver, and temporary close with negative pressure dressing. Surgical notes described a “raw appearance of liver capsule and bleeding from area of ruptured hemangioma.” The patient was then taken to interventional radiology where he had an hepatic angiogram and gel-foam embolization of the right hepatic artery. He was then extubated after stabilization with great recovery and subsequently downgraded from the intensive care unit.

## DISCUSSION

Hepatic hemangiomas, the most common benign tumors of the liver, are typically small structures less than 4 centimeters (cm) in diameter and found incidentally on imaging. These tumors are composed of atypical masses of blood vessels, and most are asymptomatic.^3^ Spontaneous rupture is an extremely rare complication with most cases being secondary to traumatic events, although very rarely it can be associated with anticoagulation administration. Rupture often presents with a combination of abdominal pain and hemorrhagic shock.^3^ Hemangiomas larger than 4 cm are considered “giant,” and a retrospective cross-sectional study showed a higher potential chance of rupture when they have exophytic characteristics and are peripherally located.^4^

Whether spontaneous or traumatic, hepatic hemangioma ruptures require emergent intervention with mortality rates approaching 37%.^5^ Surgical management consists of an exploratory laparotomy with prompt control of bleeding along with postoperative angiography.^5^ Efforts between surgery and transcatheter arterial embolization depend on hemodynamic stability and the status of the patient during symptomatic presentation. Upon literature review, we found that most cases ultimately require surgical intervention, although there have been milder cases where bleeding was managed solely with transcatheter arterial embolization.^6^,^7^

One of the limitations that must be discussed in this case involves the ultrasonographic portion of finding the active liver bleed in real time. There is a vast difference in sonographic skills between medical students, postgraduate residents, and seasoned attendings who use POCUS daily and, ultimately, those who are fellowship trained. The sonographer in this case was fellowship-trained with multiple years of POCUS experience, which may have influenced and prompted them to apply the color flow modality and thus be able to catch this active bleed. Placing color flow on FAST exam is not part of routine protocol; this EP demonstrated advanced clinical expertise and the ability to identify this pathology.

Competency in performing the FAST exam can be achieved within a couple of weeks and/or months of training. Proficiency can be achieved by performing 35–75 studies with appropriate feedback and peer review of images.^8^,^9^ Therefore, with more POCUS training and exposure, sonographers can unlock further diagnostic abilities over the course of their career.

This case highlights a rare pathology and presentation, with fewer than 75 cases documented in the literature. Even fewer have involed a hemangioma less than 5 cm in diameter. This case provides a significant contribution to the small pool of documented cases and highlights the diagnostic capabilities of the FAST exam and point-of-care imaging in the hands of a skilled sonographer. Emergency physicians should be more vigilant and potentially assess for more than just free fluid during the FAST exam and look for signs of solid organ injury and other pathology in the appropriate clinical setting.

## CONCLUSION

This case serves as a reminder to emergency physicians not to anchor on the primary diagnosis and use all the tools at their disposal, including point-of-care ultrasound, to diagnosis a crashing patient. While the use of the FAST exam is well established in the setting of trauma, this case demonstrates its utility in a non-traumatic patient with undifferentiated hypotension and shock. Spontaneous rupture of a liver hemangioma from anticoagulation should be kept in the differential diagnosis if a routine CT picks up an incidental liver lesion. Ultimately, the importance of ultrasonographic competence in physicians of all specialties is imperative to improve our diagnostic abilities in emergent situations.

## Supplementary Information

Video Right upper quadrant view of a focused assessment with sonography in trauma (FAST) exam performed on hypotension patient currently on anticoagulation for pulmonary embolism. White boxed area of color flow showing active flow from liver tip area concerning for potential active liver bleed with right kidney seen to left of white boxed area.

## Figures and Tables

**Image:**
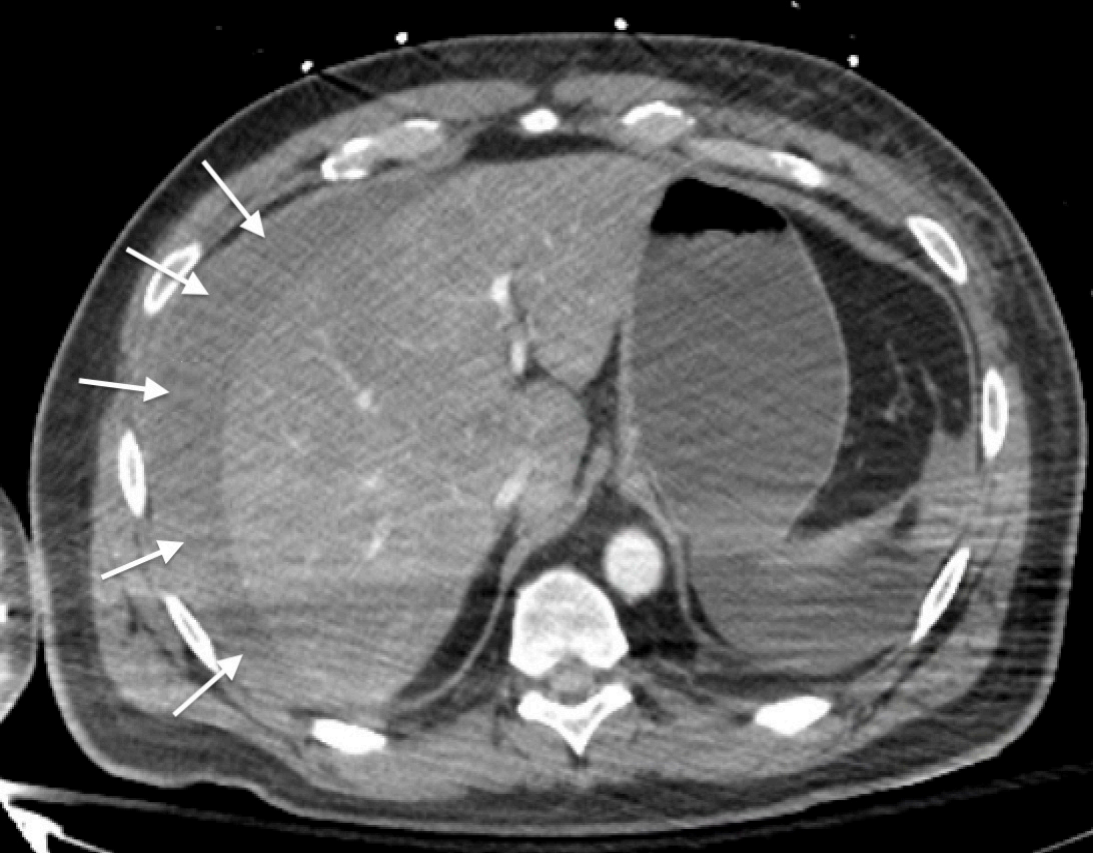
Computed tomography of abdomen and pelvis with contrast showing subscapular hyperdense blush along the right lower hepatic lobe. Favored active venous subscapular bleeding of liver with new hemoperitoneum from intraparenchymal hematoma given timing of contrast phase. Subcapsular hematoma is visible around the liver (arrows).
